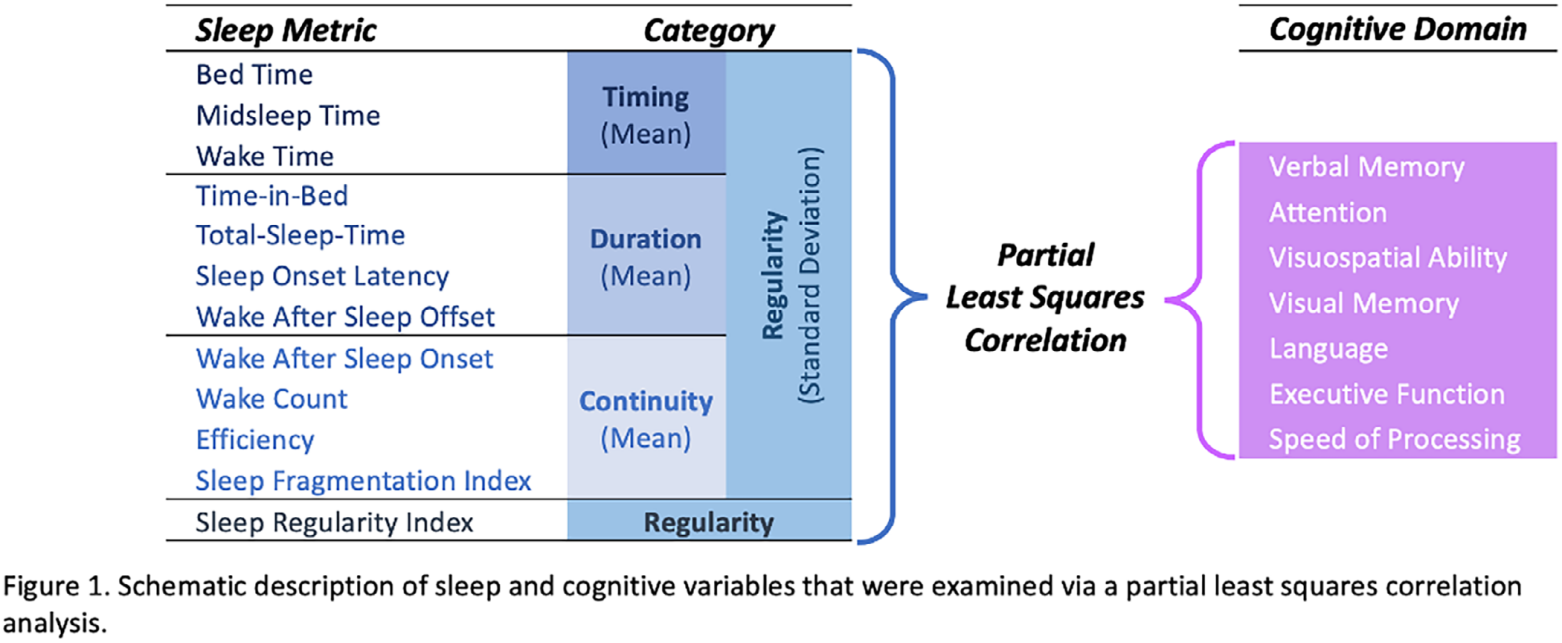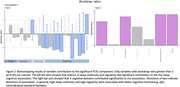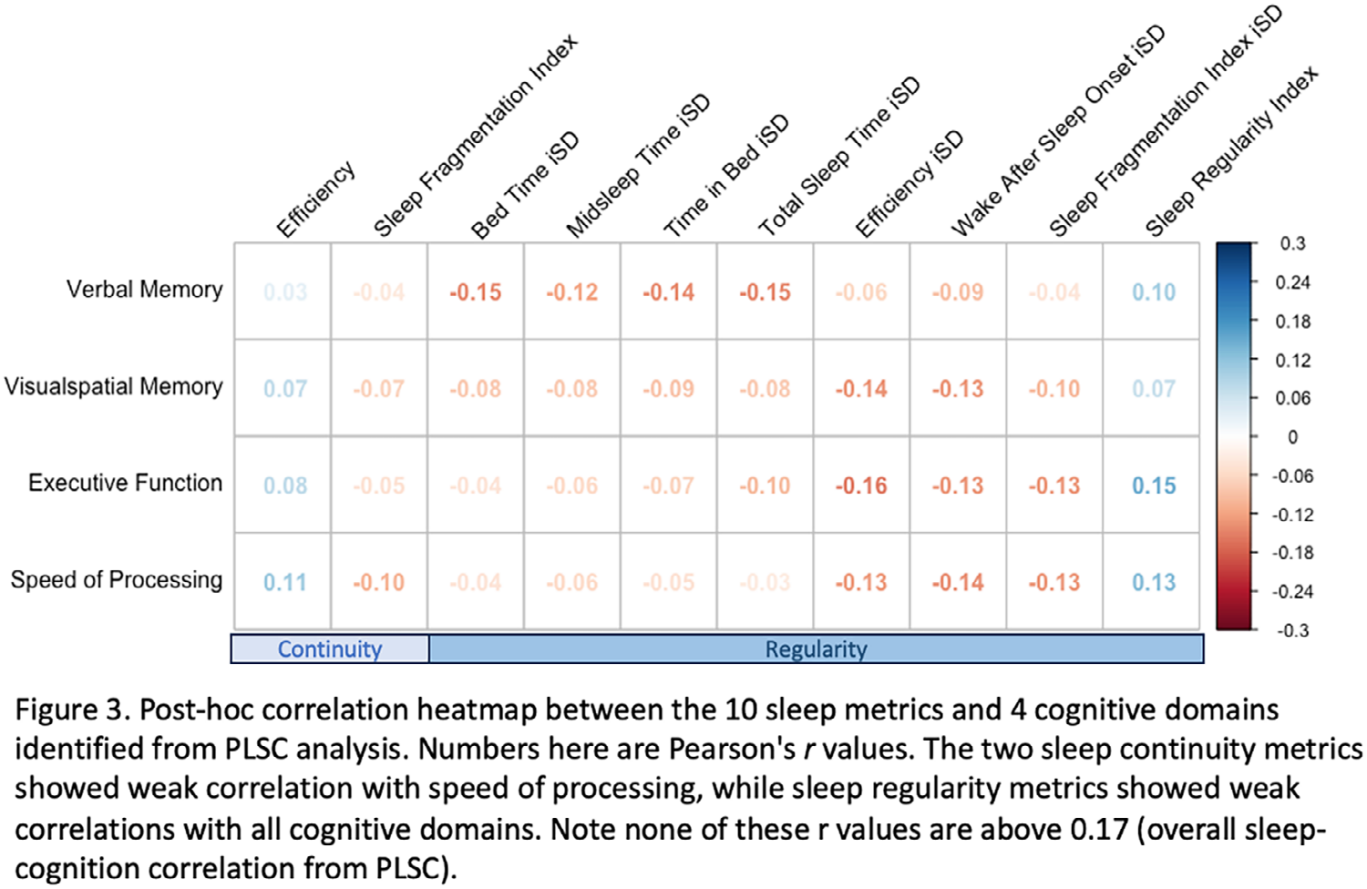# Multivariate sleep health and cognitive functions in healthy older adults: results from partial least square correlation analysis

**DOI:** 10.1002/alz.084898

**Published:** 2025-01-09

**Authors:** Shuo Qin, Eric Kwun Kei Ng, ChunSiong Soon, XinYu Chua, Juan Helen Zhou, Woon‐Puay Koh, Michael Wei‐Liang Chee

**Affiliations:** ^1^ National University of Singapore, Singapore, Singapore Singapore; ^2^ Centre for Sleep and Cognition & Centre for Translational Magnetic Resonance Research, Yong Loo Lin School of Medicine, National University of Singapore, Singapore, Singapore Singapore; ^3^ National University of Singapore, Singapore, Sinagpore Singapore

## Abstract

**Background:**

Past studies examining sleep‐cognition relationships mostly employed univariate approaches, which are subject to problems such as multicollinearity and multiple comparisons. Further, results from small sample univariate analyses are difficult to compare, precluding the identification of the aspects of sleep health associated with a particular cognitive domain(s). The current study used a multivariate approach to identify key sleep metrics and cognitive domains that contribute to the maximum sleep‐cognition covariance in healthy older adults. From the reduced list of sleep metrics and cognitive domains, novel associations between different aspects of sleep health and cognitive domains can be uncovered.

**Method:**

The current study is part of the ongoing SG70: Toward Healthy Longevity in Singapore study, which aims to comprehensively assess factors that affect aging health in over 1000 Singaporean older adults. From the SG70 study, 440 healthy older adults wore an Oura Ring (OuraHealth OY) for 14‐30 days. Twenty‐three metrics encompassing 4 major aspects of objective sleep health: duration, timing, regularity, and continuity were extracted (Figure 1). Cognition was assessed using a comprehensive battery that encompassed 7 domains. The overall covariance between sleep and cognition was examined by a partial least square correlation (PLSC) analysis. Sleep metrics and cognitive domains that contributed significantly to significant PLSC components were identified by bootstrapping.

**Result:**

PLSC analysis identified a component that explained 68% of covariance between sleep and cognition matrices (r=0.17, p<0.001). Bootstrapping tests further identified 10 sleep continuity and regularity metrics and 4 corresponding cognitive domains that contributed significantly to the observed covariance (Figure 2). Post‐hoc univariate analyses showed that sleep continuity metrics correlated with speed of processing, while sleep regularity metrics correlated with verbal memory, visual‐spatial ability, executive functions, and speed of processing (Figure 3).

**Conclusion:**

Our results suggest that sleep continuity and regularity may be more sensitive markers of impairments across multiple cognitive domains in healthy aging compared to sleep duration and timing. In addition, they support the utility of multivariate analyses in uncovering significant association patterns between sleep and cognition.